# Sign Language Dataset for Automatic Motion Generation

**DOI:** 10.3390/jimaging9120262

**Published:** 2023-11-27

**Authors:** María Villa-Monedero, Manuel Gil-Martín, Daniel Sáez-Trigueros, Andrzej Pomirski, Rubén San-Segundo

**Affiliations:** 1 Grupo de Tecnología del Habla y Aprendizaje Automático (T.H.A.U. Group), Information Processing and Telecommunications Center, E.T.S.I. de Telecomunicación, Universidad Politécnica de Madrid, 28040 Madrid, Spain; maria.villa.monedero@alumnos.upm.es; 2 Alexa AI, C. de Ramírez de Prado, 5, 28045 Madrid, Spain; dsaez@amazon.co.uk; 3 Alexa AI, Aleja Grunwaldzka 472, 80-309 Gdańsk, Poland; pomirsa@amazon.com

**Keywords:** motion dataset, sign language, sign phonemes, HamNoSys, landmarks extraction

## Abstract

Several sign language datasets are available in the literature. Most of them are designed for sign language recognition and translation. This paper presents a new sign language dataset for automatic motion generation. This dataset includes phonemes for each sign (specified in HamNoSys, a transcription system developed at the University of Hamburg, Hamburg, Germany) and the corresponding motion information. The motion information includes sign videos and the sequence of extracted landmarks associated with relevant points of the skeleton (including face, arms, hands, and fingers). The dataset includes signs from three different subjects in three different positions, performing 754 signs including the entire alphabet, numbers from 0 to 100, numbers for hour specification, months, and weekdays, and the most frequent signs used in Spanish Sign Language (LSE). In total, there are 6786 videos and their corresponding phonemes (HamNoSys annotations). From each video, a sequence of landmarks was extracted using MediaPipe. The dataset allows training an automatic system for motion generation from sign language phonemes. This paper also presents preliminary results in motion generation from sign phonemes obtaining a Dynamic Time Warping distance per frame of 0.37.

## 1. Introduction

Sign language datasets play a crucial role in developing systems that enable effective communication for individuals with hearing impairments. While several sign language datasets exist, they are focused on sign language recognition and translation, not including information at the phoneme level. Many existing datasets rely only on specific sources such as speech or text descriptions attached to videos, which fall short of capturing the intricate details inherent in sign languages. The absence of phonemes does not allow for the development of motion generation systems for sign language.

In other fields, different to sign language processing, there exist several motion datasets describing different human activities. For example, the Human3.6M dataset [1] contains 3.6 million accurate 3D human poses under four different viewpoints and their corresponding images. This dataset contains typical human activities such as taking photos, posing, eating, or talking on the phone performed by 11 professional actors. Some examples of the annotations in the dataset are “a person waves with left hand” and “the person is walking in a circular shape”.

Other datasets combine natural language annotations and gesture representations to train systems able to generate avatar motion. For instance, the KIT Motion-Language dataset [2] contains 3911 gestures, with a total duration of 11.23 h, and 6278 annotations in natural language that contain 52,903 words. The authors converted the marker-based motion capture data to the Master Motor Map framework representation (avatars). To obtain motion annotations in natural language, they applied a crowd-sourcing approach and a web-based tool called Motion Annotation.

The HumanML3D dataset [3] consists of 14,616 3D human motion clips and 44,970 text descriptions, covering a vocabulary of 5371 distinct words and a total duration of 28.59 h. This dataset covers a wide range of body movements and postures. Some examples of the text descriptions are “a person sits down and crosses their legs, before getting up” or “a person stretches arms out and makes arm circles”.

NTU RGB+D 120 [4] is a large-scale dataset for RGB+D human action recognition, collected from 106 distinct subjects, that contains more than 114 thousand video samples and 8 million frames. This dataset contains 120 different action classes, including daily, mutual, and health-related activities. These action classes cover labels such as “move heavy objects”, “pushing other person”, “arm circles”, and “squat down”.

The BABEL dataset [5] provides action labels per frame for natural and continuous human movement data and contains 43.5 h of recording from 252 action categories. These action categories cover labels such as “stand”, “run”, “support body with right hand”, and “jump over obstacle”.

Regarding datasets related to sign language, Word-Level American Sign Language (WLASL) is the largest video dataset for ASL recognition [6], including 2000 common different words performed over 100 signers. This dataset has been exploited to recognize signs but also to generate 2D human pose representations using OpenPose [7]. Another dataset, called How2Sign [8], included speech and transcriptions of videos. This dataset contained a 16k English words vocabulary and became a rich set of annotations including gloss, category labels, as well automatically extracted 2D keypoints for more than 6M frames. The LSE-Sign database [9] includes Spanish Sign Language information, including 2400 individual signs as well as grammatical, phonological, and articulatory information. Other studies combine different types of sensors for sign language recognition [10]. However, these datasets do not include both sign phonemes and sign motion landmarks, preventing the training of an automatic system with the sufficient level of detail to generate sign language motion from sign characteristics. These datasets have been traditionally used for sign language recognition [11].

In this paper, we introduce a new sign language dataset that addresses this limitation by incorporating phoneme representations for each sign. By providing these phonemes for each sign, we bridge this gap and unlock new possibilities for sign language motion generation with enough precision. The main contributions of this paper are as follows:

- The first sign language dataset for automatic motion generation, including sign videos and the corresponding phonemes in HamNoSys. HamNoSys is a transcription system for any sign language and was developed at the University of Hamburg, Hamburg (Germany).
- A detailed description of the methodology for generating the dataset: phonemes and motion information.
- A strategy for landmarks extraction from sign language videos. This strategy includes the use of MediaPipe for combining pose and hand landmarks. A solution is provided for dealing with coherence problems during the landmark extraction process along the frame sequence of a sign.
- Finally, the paper presents preliminary experiments for automatic motion generation from sign language phonemes using state-of-the-art deep learning algorithms based on transformers.

The motivation behind this research arises from the need to fill a gap in sign language dataset generation. By introducing the first dataset for automatic motion generation, encompassing phonemes and motion information, this study aims to contribute to the advancement of sign language research. Furthermore, the exploration of preliminary experiments using state-of-the-art transformers for generating motion from these sign language phonemes serves as a driving force to expand the frontiers of automatic motion generation in sign language applications.

This paper is organized as follows. Section 2 describes the dataset generation process, including details about phoneme information, the videos, and the landmarks. Section 3 contains the methods used to generate sign language motion based on landmarks from sign characteristics through a transformer-based architecture. Section 4 discusses the main contributions of the paper. Finally, Section 5 summarizes the main conclusions of the paper.

## 2. Dataset Generation

This dataset includes Sign Language content, including sign phonemes (HamNoSys), sign videos and landmarks sequences including relevant body points: face, arms, hands and fingers. This dataset includes 754 different signs of Spanish Sign Language (LSE: Lengua de Signos Española) represented by three different subjects with three different camera orientation. The MediaPipe library [12] has been used to extract the landmarks information from the videos. This dataset includes the information of a total of 6786 examples of signs.

### 2.1. Sign Language Vocabulary including Phoneme Information

**Figure 1:**
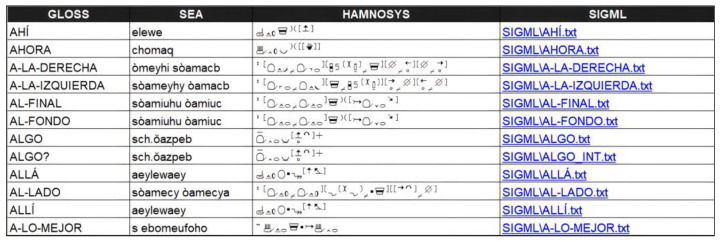
Partial view of the sign database [14].

The first step has been the creation of the sign language vocabulary including the most frequent sign using in LSE: the entire alphabet, numbers from 0 to 100, numbers for hour specification, months, weekdays, greetings, etc. This vocabulary includes 754 different signs. For each sign, we have included detailed information: gloss (word in capital representing the sign); phoneme information in SEA (Sistema de Escritura Alfabética) [13]; phoneme information in Hamburg Notation System (HamNoSys); and the corresponding Signing Gesture Markup Language (SiGML) information, which is an XML (eXtensible Markup Language) representation of HamNoSys. Figure 1 shows a partial view.

The phoneme information has been generated using a new version of eSign Editor, improving the one developed in the VISICAST, eSign, and DictaSign European Projects [14,15,16]. This new version incorporates a SEA-HamNoSys converter. SEA is an alphabet (based on ASCII characters) for sign language. SEA allows a sequence of characters to be specified that describe aspects such as hand shape, hand position, location, and movements. The reason for developing this converter is that the first normative dictionary for LSE (developed at Fundación CNSE: http://www.fundacioncnse.org/tesorolse (accessed on 1 October 2023)) has SEA descriptions for more than 4000 signs, and with this converter was possible to automatically generate HamNoSys information from SEA descriptions. Additionally, we have included the HamNoSys description in SiGML. SiGML preserves the same information as the HamNoSys description of a sign but is designed to be processed by computers, providing a language that computers can understand and work with. This is the information we will use for training a motion generation system.

**Figure 2:**
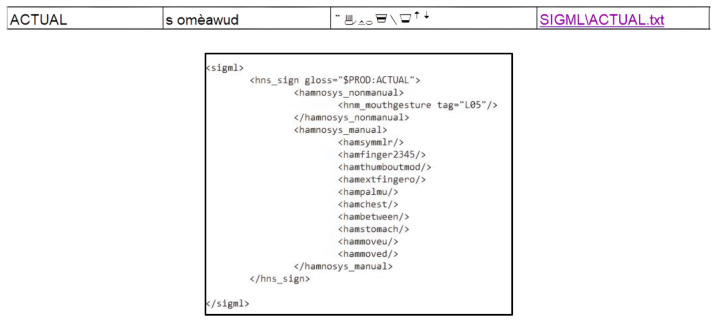
HamNoSys to SiGML for the sign ACTUAL.

Figure 2 illustrates an example of the “translation” from HamNoSys to SiGML representation for ACTUAL sign.

As observed in Figure 2, in SiGML notation, we observed two types of tags: <hamnosys_nonmanual> and <hamnosys_manual>. While HamNoSys primarily focuses on the manual aspect of signs, the inclusion of non-manual components such as eye movements, head nods, mouth expressions, and others is crucial in sign language.

HamNoSys is a phonetic transcription system for sign language, which includes more than 200 symbols. HamNosys was developed at the Institute of German Sign Language at the University of Hamburg, for transcribing individual signs and phrases in sign language. HamNoSys has been designed considering the basic principles described below [17]:

- Sign Language independence. HamNoSys exhibits a universal quality that transcends specific sign languages, enabling the description of signs from any sign language. This characteristic originated from the primary objective of developing HamNoSys as a tool for researchers to articulate sign descriptions.
- Focus on posture and movement. HamNoSys captures the physical positioning and motion of hands. While a sign representation may possess varying interpretations in different contexts, its HamNoSys transcription remains the same. This aspect is crucial for motion generation.
- Omission of irrelevant information. HamNoSys exclusively delineates the relevant elements of posture and movement crucial for sign formation. HamNoSys focused on hands and face, omitting factors such as shoulder or elbow positioning because they do not affect the sign meaning.

**Figure 3:**
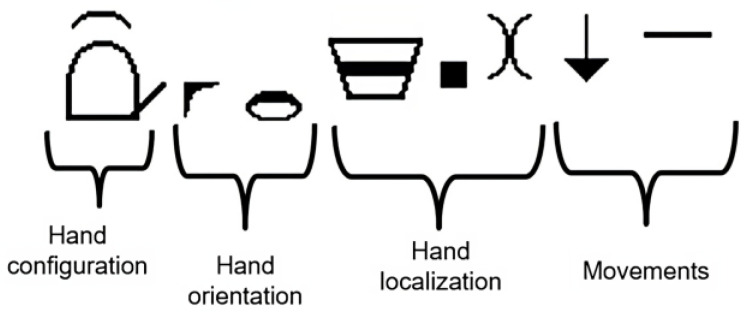
Main parts of a sign description in HamNoSys [17].

HamNoSys describes the signs in terms of hand shape, orientation, position, and movements, as shown in Figure 3. Every aspect is considered as a phoneme in sign language.

- Hand shape: The HamNoSys system defines 12 standardized forms that represent different hand shapes. Additionally, HamNoSys facilitates the specification of the extended finger by incorporating the appropriate symbol (ranging from 1 to 5) to indicate the finger being extended.
- Hand orientation: Hand orientation information is divided into two parameters within HamNoSys. The first parameter refers to the direction of the base of the forefinger, which signifies the hand’s axis orientation. The second parameter encompasses the palm’s orientation, which determines the hand’s alignment along the axis.

The extended finger direction (EFD) corresponds to the direction that would be indicated by the extended finger, offering a total of 26 feasible representations. 

Furthermore, the orientation of the palm is characterized by eight distinct values, indicating various orientations relative to the hand’s shaft.

- Hand position: Within HamNoSys, a set of symbols exists to describe the positioning of the hand relative to the body. This includes the indication of contact between the hand and specific body parts, as well as intermediary positions between two defined symbols.
- Hand movements: Describing hand movements within HamNoSys can be complex. HamNoSys accommodates the definition of hand movements in spatial terms, as well as movements that do not alter the hand’s location.
- Regarding the hand movements through space, the following categories are recognized: straight, curved, circular, or movements aiming at a particular location. Each have multiple representations denoting distinct movement types. On the other hand, movements without changing the hand’s placement may refer to changes in the shape or orientation of the hand, movement of the wrist, and movement of fingers.

### 2.2. Videos Generation

To depict the sign language representations, we used a software program developed in Visual C++ [17] that allows users to specify one of three available avatars to embody the sign language expressions. This software incorporates several avatars developed in the eSIGN and DictaSign [18] European projects [16,19] as an ActiveX control. This control includes a software library that contains all the necessary methods for animating and virtualizing the avatar. This software has been developed by the Virtual Humans Group in the School of Computing Sciences at University of East Anglia [20] and it can be used on terms equivalent to Creative Commons BY-ND, allowing for its use in research. Some significant methods are:

- GetFrameRate(): Yields the rate of frames per second set to represent a sign;
- SetFrameRate (short nNewValue): Sets a rate of frames per second for the representation of the signs;
- Initialise (long t): Loads the corresponding animated avatar;
- PlaySiGML (LPCTSTR bsSIGMLIn): The animated avatar represents the signed described in the file;
- SwitchAvatar (long t): Lets you change the displayed animated avatar.

The Visual C++ program depicts each sign (from the SiGML description) using the specified avatar, generating a separate video for each represented sign. These videos are then divided into frames, and the resulting files are saved locally in avi format for the videos and bmp format for the frames. 

**Figure 4:**
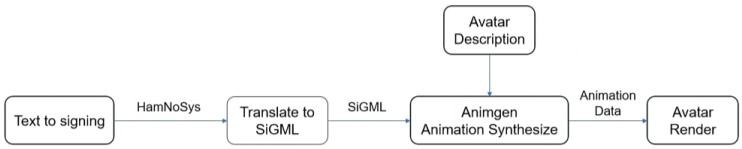
Block diagram of the video generating video process. Adapted with permission from ref. [17] Copyright 2010 Rubén San-Segundo.

Figure 4 displays the block diagram illustrating the process of generating the target database.

As a result of the entire process described above, a comprehensive dataset is obtained comprising a total of 6786 videos featuring 754 different words and sets of words represented through sign language by three distinct avatars from three viewpoints. Within this dataset, one-third of the videos feature the avatar facing forward, while the remaining two-thirds feature the avatars facing to the right and left. For each video generated we have obtained a frame periodically, resulting in an average of 21 frames per sign. 

**Figure 5:**
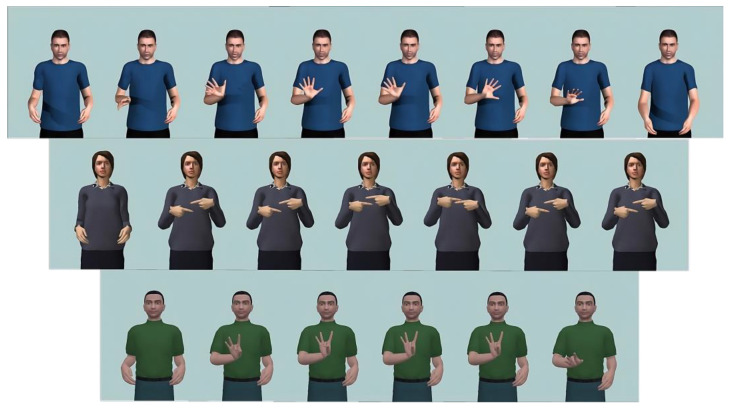
Examples of three signs and three avatars of the dataset: “Adiós”, “Familia”, and “Sí” glosses, which mean “Goodbye”, “Family” and “Yes”, respectively.

Figure 5 shows examples of three signs included in the dataset, including representative frames from each sequence.

### 2.3. Landmarks Extraction Using MediaPipe

In this work, we have used MediaPipe software (version 0.9.0.1) to extract the x, y, and z coordinates of 75 pose and hand landmarks from each frame. All the coordinates have values in the [0.0, 1.0] interval, considering the height and width of the input images. 

#### 2.3.1. Pose and Hand Detection

**Figure 6:**
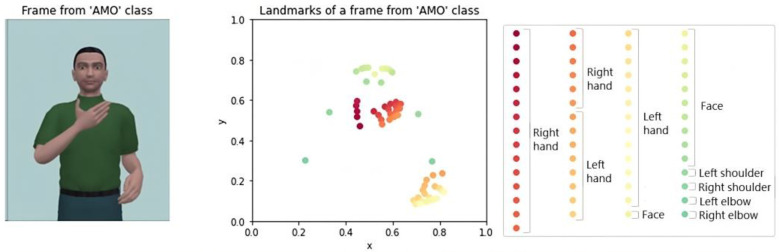
Frame from a sign chosen for this study and its landmarks (red ones are related to right hand, orange and yellow ones are related to the left hand, and green ones are related to the body).

The MediaPipe library uses independent models to detect pose and hands landmarks: 33 landmarks from the pose (MediaPipe Pose) and 21 landmarks from each hand (MediaPipe Hands). The 33 landmarks from the pose arrange several locations of the body such as face, shoulders, hip and upper and lower limbs (including elbows, knees, hands, and feet). The 21 landmarks represent various locations on the hand, including the wrist and four points along each of the five fingers. Since the avatar is visible in a 2D video from the waist up, the software offers values for z coordinates and non-visible landmarks based on body constraints models. Moreover, we decided to reduce the total number of landmarks from 75 to 57 because some of those could be redundant or less informative. This way, we keep the 42 landmarks obtained from MediaPipe Hands and 11 face landmarks and the ones from the shoulders and elbows from MediaPipe Pose. Figure 6 illustrates a frame and their landmarks for pose and hands.

It was necessary to use MediaPipe Pose to extract information for the whole body and determine the posture while signing. However, since this model does only extract a few key points for the hands, it is crucial to use MediaPipe Hands to increase precision in the fingers motion, which determines the meaning of the different signs.

#### 2.3.2. Problem: Detecting Mirrored Hands 

MediaPipe Hands software is specifically designed to detect landmarks from the hands, extracting information from each hand independently and labelling each point belonging to one of the hands. In this process, it was necessary to deal with several extraction problems. However, this model occasionally fails to properly detect the hand landmarks and ends up misidentifying the hands. The main problem is the detection of the hand being mirrored. MediaPipe may mistakenly assign the landmarks from the right hand to the left hand and vice versa. This issue can occur due to several factors, such as lighting conditions, occlusion, or variations in hand appearances in specific frames. As a result, the mislabeled hand landmarks obtained from MediaPipe Hands generate incoherent hand movements that could lead to inaccurate interpretations or actions based on the detected hand gestures.

**Figure 7:**
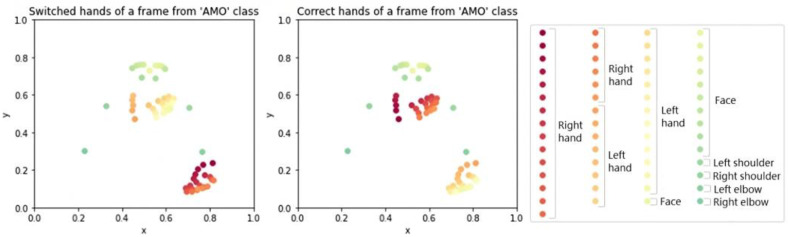
Switched landmarks for the hands (left) before applying the correction algorithm to obtain the correct landmarks for the hands (right).

For this reason, the solution proposed in this work involves complementing MediaPipe Hands model with MediaPipe Pose model. The Pose model includes landmarks around the whole body, including the wrists. This model does properly detect the landmarks of the right and left wrist since it has the whole body as a reference (including the detection of the face oriented to the camera). This way, we have implemented a correction algorithm that computes the distance between each wrist landmark obtained from MediaPipe Hands and MediaPipe Pose. Considering the minimum distance, it is possible to determine which hand the landmarks belong to. In case of a mismatch between the original labelling from MediaPipe Hands and the actual position, the mislabeled landmarks must be relabeled based on the correct hand detected by MediaPipe Pose. Figure 7 displays an example of mislabeled landmarks for the hands and the result after applying the correction algorithm.

By incorporating this solution, we took advantage of the strengths of both models and leveraged the contextual information provided by the pose detection model to rectify the errors in the hand landmark detection model. Therefore, we assured that all the extracted landmarks provide an accurate location, removing the ambiguity of original landmarks.

### 2.4. Final Statistics

In conclusion, our dataset comprises 6786 videos, with an average of 21 frames per video. To create this dataset, we used a total of 754 glosses. 

Table 1 displays the different orientations of the avatars, along with the number of signs represented through the videos and the number of frames per video. An example of the dataset is included in the Supplementary Materials.

## 3. Sign Language Motion Generation

Since the original Visual C++ solution is excessively labor-intensive, we have used the dataset described above to automatically generate motion from the sign phonemes in HamNoSys (using SiGML notation) using novel deep learning techniques. This developed system will be able to generate the motion information associated with a combination of sign characteristics not seen in the training process. This aspect is an important advantage compared to the original solution.

**Figure 8:**
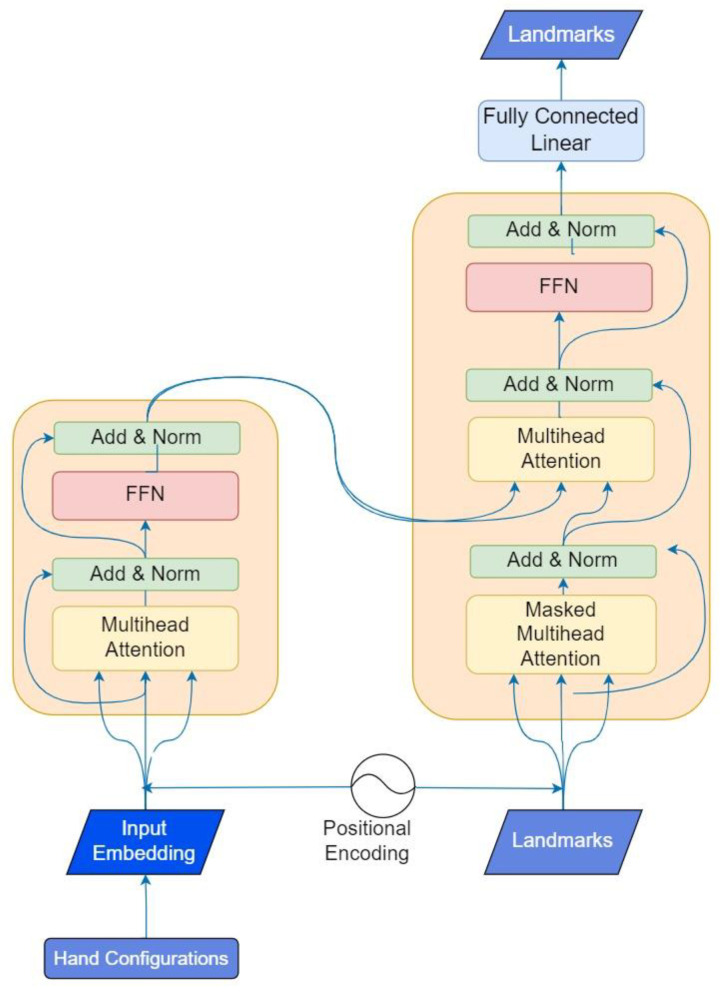
Transformer architecture.

To accomplish the generation of motion, we employed a transformer-based architecture inspired by a prior study [21] that primarily focused on speech synthesis. However, in our case, we have made specific adaptations to suit the requirements of our encoder and decoder modules, which involved translating sign language characteristics into motion, i.e., sequence of landmarks. The encoder inputs were the SiGML representations, which were tokenized using the WordPiece Tokenization Algorithm, used by BERT [22], that splits the text (SiGML representation of HamNoSys) into smaller units called tokens [23]. The encoder receives sign phonemes and uses self-attention [24] and feed-forward neural networks for processing the input information. Through self-attention, the model assesses sign characteristics in the input data, highlighting the most pertinent ones for each output. Regarding the decoder inputs, they were the MediaPipe landmarks (x and y coordinated from the 57 key points around the body and hands). This decoder requires a PositionalEmbedding layer to learn a position embedding for the inputs (previously generated landmarks) and generates an output sequence of landmarks based on the sign phonemes (encoder inputs) and previously generated landmarks. Figure 8 shows a structure of the transformer architecture. Since the signs in the dataset had different duration, we decided to fix a maximum length of frames for all the examples at 80 frames. This way, we performed zero-padding to include zeros in the landmarks representation until reaching this maximum length. The Mean Absolute Error (MAE) loss was used in the last layer, providing a measure of the absolute difference between the predicted and ground truth landmark coordinates.

The whole system was programed in Python 3.8.10 using Tensorflow 2.12.0 and Keras 2.12.0.

### Preliminary Experiment

In this work, we have conducted a preliminary motion generation experiment using 2262 videos from the avatars facing forward. We have considered a k-fold cross-validation methodology (k = 5) in which the data was divided into k groups or folds to train, validate, and test the system with different data. 

For evaluating the quality of the system, we have used the DTW (Dynamic Time Warping) metric. This metric finds the minimum mapping accumulated distance between the landmarks of two frame sequences (ground truth and predicted) divided by the length of the sign (in frames). This metric needs to compute the distance between the landmarks of two frames using Equation (1), where the subscripts “O” and “P” indicate the original and predicted frames, respectively, and the subscript “i” denotes the landmark number. In this work, we used the DTW obtained from the dtaidistance.dtw_ndim.distance Python function [25].

(1) $$DTW\left(O,P\right)=\sum_{i=0}^{N}\sqrt{{\left(x_{Oi}-x_{Pi}\right)}^{2}+{\left(y_{Oi}-y_{Pi}\right)}^{2}}$$

The generated dataset was used to train a transformer responsible for generating landmarks from sign representations. In the preliminary experiment, the transformer achieved a DTW value of 0.37 ± 0.23, as shown in Table 2. 

As potential future research, it could be possible to refine the proposed deep learning architecture based on transformers including a stop detection module to determine the end of the motion generation process. In addition, other padding strategies or data augmentation techniques could be explored to enhance the system performance. Moreover, since the evolution of the signs was represented through the landmarks of consecutive images, it would be interesting to apply interpolation to smooth the associated motion. These future directions have the potential to advance the effectiveness and efficiency of the sign language motion generation system.

## 4. Discussion

In this study, we present the first sign language dataset tailored specifically for automatic motion generation. This dataset comprises sign videos accompanied by their corresponding phonemes transcribed in SiGML notation. The creation of this dataset is rooted in the necessity for comprehensive resources to advance the field of sign language research and application.

In this paper, we have provided a detailed description of the processes involved in capturing both phonemes and motion information, allowing for the creation of a complete dataset that captures the intricacies of sign language expression. Table 3 shows a comparison of the previous works, focused on sign language dataset creation, with the dataset generated in this work. As it is shown, our dataset is the only one that includes both sign phonemes and landmarks related to the sign motion.

An important aspect of our dataset development is the strategy employed for landmarks extraction from sign language videos. Leveraging MediaPipe for the fusion of pose and hand landmarks, we have introduced a systematic approach to address coherence problems that may arise during the landmark extraction process along the frame sequence of a sign.

The significance of our dataset has been also supported by preliminary experiments in automatic motion generation. We explored the potential of our dataset in training a transformer-based model for generating sign language motions automatically. These experiments serve as an example of the practical utility and innovation of our dataset.

## 5. Conclusions

This work introduces a new sign language dataset for motion generation including sign phonemes in HamNoSys. The whole process has been explained and detailed. This paper also outlines the methodology employed for extracting landmarks from videos featuring sign language. The dataset comprises signs performed by three avatars comprising a total of 754 different signs. This dataset includes a collection of 6786 videos, each accompanied by corresponding phonemes (HamNoSys annotations) that describe the signs. Additionally, the dataset includes landmarks extracted from every video frame, including pose and hands. In this paper, a problem regarding the detection of mirrored hands by MediaPipe has been detected, providing a solution. The solution consisted of using the wrist landmarks from the pose extractor as a reference for the hand extractor. The goal is to detect if the landmarks from a hand belong to the right or left hand.

The paper also reports preliminary results in motion generation for generating landmarks from sign representations. The preliminary experiment showed a DTW value of 0.37 ± 0.23.

Regarding limitations of the proposed method, MediaPipe could have problems extracting landmarks from blurred images, images without complete hands or images where the definition of the avatar is not completely clear.

For future work, we are considering using this methodology to create a bigger dataset including a higher number of signs from real videos. Moreover, we would like to deeply analyze the possibilities to create more robust transformer architecture to reduce the DTW metric for the sign language generation.

## Figures and Tables

**Table 1:** Final statistics of the dataset.

Avatar Orientation	Number of Signs	Number of Frames per Video (Average)	Number of Videos per Sign	Number of Generated Videos
Forward	754	19	3	2262
Left	754	19	3	2262
Right	754	24	3	2262
Total	754	21	9	6786

**Table 2:** DTW mean and standard deviation values for generating sign language motion.

	DTW
Mean	0.37
Standard Deviation	0.23

**Table 3:** Characteristics of previous dataset focused on sign language (in bold the dataset described in this paper).

Dataset	Number of Signs	Number of Videos	Sign Phonemes	Motion Landmarks	Number of Orientations of Signers
WLASL2000 [6]	2000	21,083	No	No	1
How2Sign [8]	16,000	35,191	No	Yes	3
LSE-sign [9]	2400	2400	Yes	No	2
**New dataset**	**754**	**6786**	**Yes**	**Yes**	**3**

## Data Availability

The dataset can be obtained by requesting it via email to the corresponding author.

## Supplementary Materials

The following supporting information can be downloaded at: https://www.mdpi.com/article/10.3390/jimaging9120262/s1.
