# Supplementary figures and images for: Sign Language Dataset for Automatic Motion Generation
Source: J Imaging. 2023 Nov 27;9(12):262. doi: 10.3390/jimaging9120262 (PMC10744067; doi:10.3390/jimaging9120262)

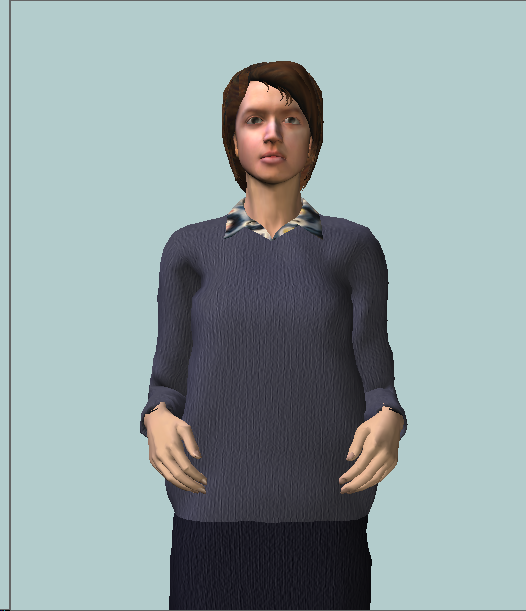

Supplement: Supplementary file 1 [file jimaging-09-00262-s001.zip › A-PARTIR-DE-AHORA_10.bmp]

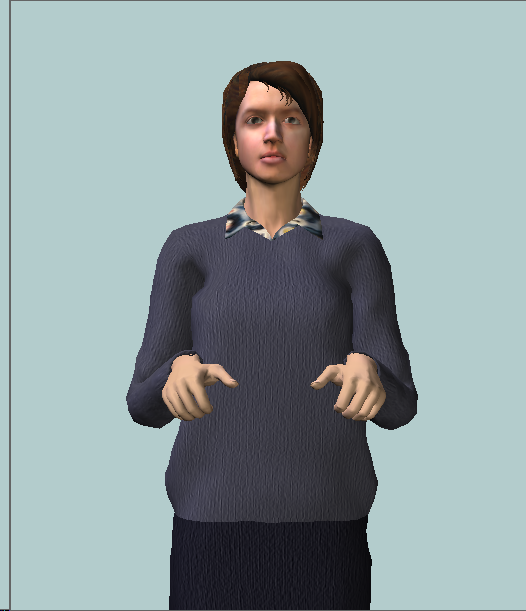

Supplement: Supplementary file 1 [file jimaging-09-00262-s001.zip › A-PARTIR-DE-AHORA_11.bmp]

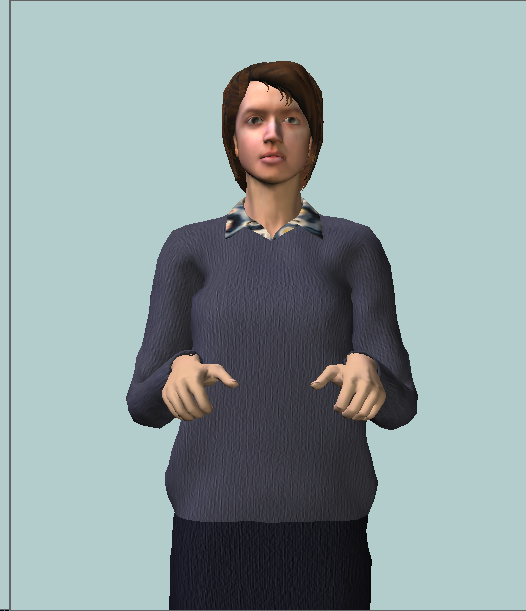

Supplement: Supplementary file 1 [file jimaging-09-00262-s001.zip › A-PARTIR-DE-AHORA_12.bmp]

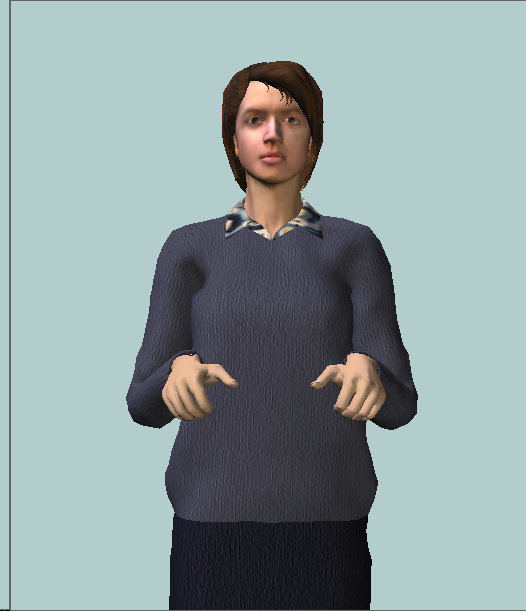

Supplement: Supplementary file 1 [file jimaging-09-00262-s001.zip › A-PARTIR-DE-AHORA_13.bmp]

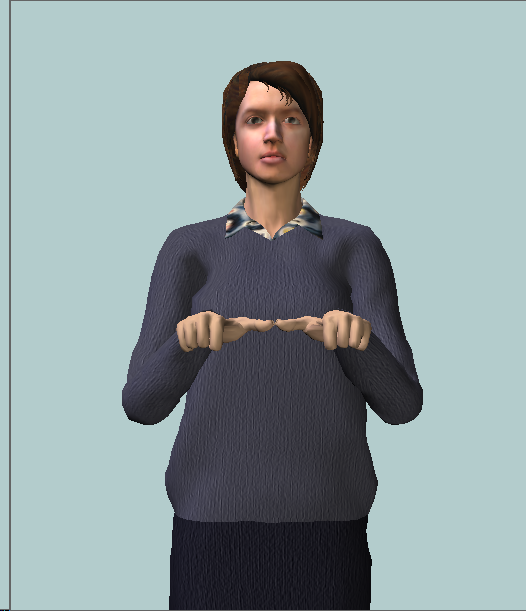

Supplement: Supplementary file 1 [file jimaging-09-00262-s001.zip › A-PARTIR-DE-AHORA_14.bmp]

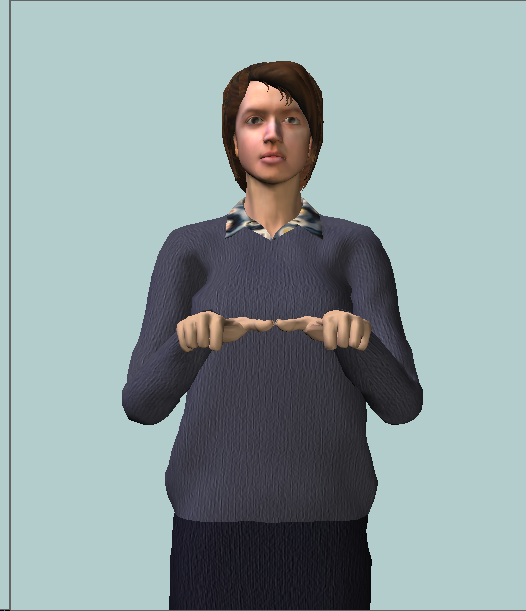

Supplement: Supplementary file 1 [file jimaging-09-00262-s001.zip › A-PARTIR-DE-AHORA_15.bmp]

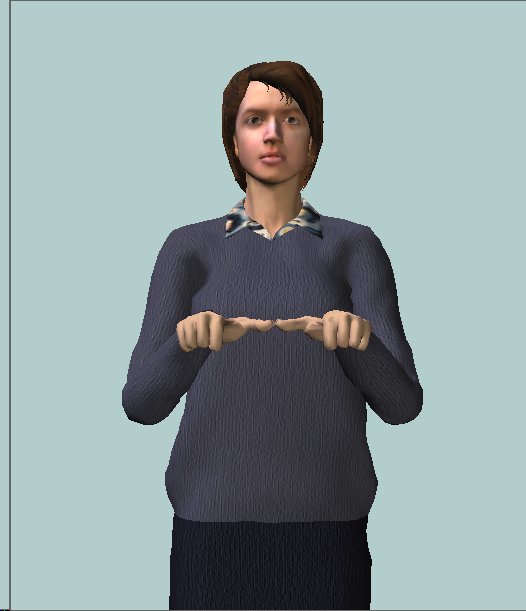

Supplement: Supplementary file 1 [file jimaging-09-00262-s001.zip › A-PARTIR-DE-AHORA_16.bmp]

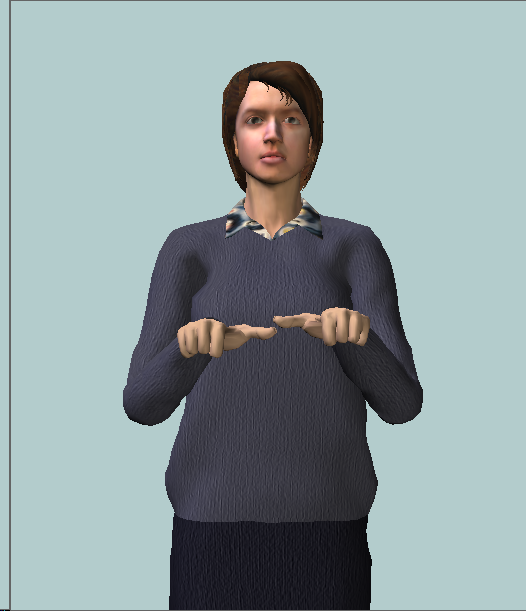

Supplement: Supplementary file 1 [file jimaging-09-00262-s001.zip › A-PARTIR-DE-AHORA_17.bmp]

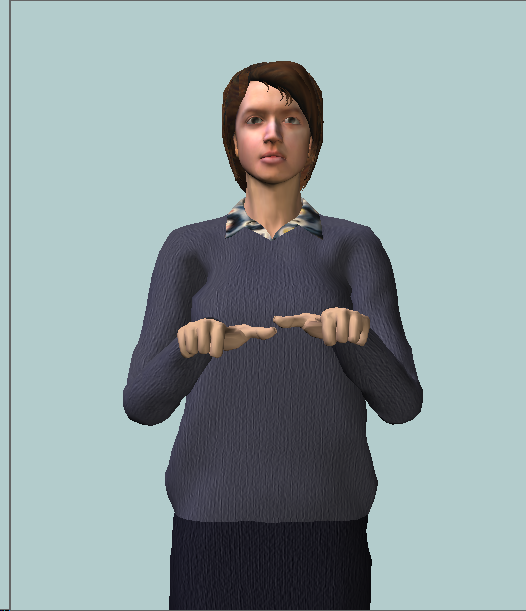

Supplement: Supplementary file 1 [file jimaging-09-00262-s001.zip › A-PARTIR-DE-AHORA_18.bmp]
